# Associations of Dietary Fat Intake With Mortality From All Causes, Cardiovascular Disease, and Cancer: A Prospective Study

**DOI:** 10.3389/fnut.2021.701430

**Published:** 2021-08-09

**Authors:** Xiaolin Yao, Xin Xu, Shuo Wang, Dan Xia

**Affiliations:** Department of Urology, The First Affiliated Hospital, Zhejiang University School of Medicine, Hangzhou, China

**Keywords:** fatty acids, mortality, cardiovascular disease, cancer, cohort

## Abstract

The impact of fat intake on health has become a growing public concern. The existing evidence linking specific dietary fat intake with mortality is controversial. We aimed to investigate the association between fat intake and total and cause-specific mortality in the Prostate, Lung, Colorectal, and Ovarian (PLCO) cancer screening trial. Intakes of saturated fatty acids (SFAs), trans-fatty acids (TFAs), monounsaturated fatty acids (MUFAs), and polyunsaturated fatty acids (PUFAs) were assessed *via* food frequency questionnaires. The primary outcomes were total, cardiovascular disease (CVD), and cancer mortality. Multivariable hazard ratios (HRs) and 95% confidence intervals (CIs) were estimated using Cox regression model adjusting for confounders. Overall, 24,141 deaths were recorded over a total 1,672,715 person-years of follow-up. There was a significant positive association between SFA consumption and total mortality (HR_*Q*5 *vs*. *Q*1_ = 1.13, 95% CI 1.05–1.22; *P*_for trend_ < 0.001). PUFA intake was strongly inversely associated with total mortality (HR_*Q*5 *vs*. *Q*1_ = 0.79, 95% CI 0.73–0.85; *P*_for trend_ < 0.001) and CVD mortality (HR_*Q*5 *vs*. *Q*1_ = 0.66, 95% CI 0.58–0.75; *P*_for trend_ < 0.001). There was a similar, but to a lesser extent, association between MUFA intake and total and CVD mortality [HR_*Q*5 *vs*. *Q*1_ 0.91 (95% CI: 0.84–0.99), *P*_for trend_ = 0.044 and 0.85 (0.73–0.98), *P*_for trend_ = 0.020, respectively]. None of these types of dietary fat were associated with cancer mortality (all *P*_for trend_ > 0.05). In conclusion, this study observed a detrimental effect of SFA intake on total mortality; in contrast, greater consumption of PUFAs and MUFAs were associated with lower risks of all-cause death and CVD mortality.

## Introduction

Quality rather than quantity of dietary fats have been emphasized at least for a decade now, and emerging studies have found that different types of dietary fats have divergent effects on health (1). Results from relatively old meta-analyses failed to find an association of saturated fatty acids (SFAs) with death from any cause or from cardiovascular disease (CVD) mortality (2, 3). Conflicting results were obtained in a recent large meta-analysis based on prospective studies where higher dietary intake of SFAs was significantly associated with a greater risk of CVD mortality (4). Polyunsaturated fatty acids (PUFAs) were reported to be associated with a lower risk of CVD and mortality in most observational studies and clinical trials (5, 6). However, there were also some studies that did not support a significant relationship between PUFA intake and all-cause mortality (7). Little and conflicting evidence exists to associate monounsaturated fatty acid (MUFA) intake with risk of mortality. One possible reason is that dietary MUFAs come from both plant- and animal-derived food with divergent dietary components that may have different effects on health outcomes. MUFAs from plant (P-MUFAs) were reported to be inversely associated with total mortality, whereas MUFAs from animal (A-MUFAs) were associated with higher mortality (8). Few epidemiological studies have focused on the effect of trans-fatty acids (TFAs) intake on mortality. A recent prospective study found that a higher consumption of TFAs was associated with increased mortality risk (9).

Based on the available data, the associations between different types of dietary fat intake and mortality remain conflicting. Public concerns have been growing with regard to the effect of fat intake on health (10). To aid in guiding recommendations on optimal fat intake, we assessed the associations of major dietary fats with all-cause death, CVD, and cancer mortality in a large prospective cohort study. We hypothesized that individual types of dietary fat determine their associations with mortality in the general population.

## Methods

### Subjects and Study Design

The design and methods of the Prostate, Lung, Colorectal, and Ovarian (PLCO) cancer screening trial have been previously published (11). Briefly, the PLCO study is a large-scale clinical trial that aimed to investigate whether certain screening tests reduce death from PLCO cancer. PLCO study consisted of 154,897 eligible participants aged 55–74 years and enrolled between November 1993 and July 2001. The participants were from 10 clinical screening centers throughout the United States. PLCO cancer screening trial was approved by the institutional review boards of the National Cancer Institute and each of the participating centers. An informed consent was obtained from each eligible participant in the study. The ClinicalTrials.gov numbers for PLCO are NCT00002540, NCT01696968, NCT01696981, and NCT01696994. The approved number of our project is PLCO-587.

### Data Collection and Dietary Assessment

The baseline questionnaire (BQ) included self-reported information on demographic information, family health history, medical history, health behaviors, and other factors. Dietary data were collected using the validated PLCO Diet History Questionnaire (DHQ) version 1.0 (National Cancer Institute, 2007), which included the prespecified portion size and consumption frequency of 124 food items and supplement use over the previous year (12). The DHQ has been validated and found to be as good as or better than two commonly used food frequency questionnaires (FFQs) at the time the PLCO study was performed (12). The USDA 1994–1996 Continuing Survey of Food Intakes by Individuals (13) was used to calibrate DHQ data and calculate the daily intake of dietary fats, including total fat, SFAs, MUFAs, PUFAs, and TFAs. We also separated MUFAs into P-MUFAs and A-MUFAs according to the food sources.

### Subject Selection

Participants were excluded from this study if they had not returned a BQ (*n* = 4,918); had reported a previous cancer at baseline (*n* = 10,199); did not have follow-up time (*n* = 12); or did not complete DHQ or the DHQ was not valid (n = 37,936). Thus, the cohort for analysis consisted of 101,832 individuals.

### Outcome Assessment

Follow-up time was calculated from the date of DHQ completion to the time of death or the last time of follow-up (NCI is extending the follow-up of PLCO participants for at least 5 years). Deaths were identified by annual mailed questionnaires and periodic linkage to the National Death Index. The cause of deaths was classified according to the International Classification of Diseases, 9^th^ Revision (ICD-9). Follow-up and classification of cause of death was done centrally through the NCI. The primary outcomes of interest were total mortality (death from any cause) and mortality from CVD or cancer.

### Statistical Analysis

The dietary fat intake was first examined as quintiles. A multivariate Cox proportional hazards (PHs) model was used to estimate hazard ratios (HRs) and 95% confidence intervals (CIs). Four stepwise models were established to adjust for covariates of known or suspected risk factors for death. Model 1 was adjusted for age (continuous) and gender (male vs. female). Model 2 was additionally adjusted for race (non-Hispanic White vs. Other), body mass index (BMI, continuous), education (≤ high school vs. ≥ college), smoking status (never *vs*. former, ≤15 years since quit vs. former, >15 years since quit vs. former, year since quit unknown *vs*. current smoker, ≤1 pack per day *vs*. current smoker, >1 pack per day vs. current smoker intensity unknown), total energy intake (continuous), alcohol drinking status (never vs. former vs. current), study center (categorical), marital status (married vs. not married), randomization arm (screening group vs. control group), aspirin use (yes vs. no), history of hypertension (yes vs. no), history of diabetes (yes vs. no), vegetable intake (continuous), and fruit intake (continuous). Model 3 was further adjusted for history of stroke (yes vs. no) and history of heart attack (yes vs. no). The final multivariable Model 4 was additionally adjusted for other remaining fatty acids. If results were divergent across different models, we used the results from the most fully adjusted model. Several methods for energy adjustment were commonly performed, such as the residual method. In our study, we adjusted the total energy intake as a confounding factor in the Cox multivariable analysis, which was also widely used in the previous studies (14, 15).

Tests of multiplicative interaction were performed using likelihood-ratio tests compared models with and without the interaction term. The PH assumption was examined using the Schoenfeld residual test (16). Restricted cubic spline models (17) were fitted with three knots (i.e., 10th, 50th, and 90th percentiles) to assess the dose–response trend in the association between specific dietary fat intake (as a continuous variable) and each outcome after full adjustment. All statistical analyses were performed using the software STATA version 15 (Stata Corp, College Station, TX, United States). All tests were two sided.

## Results

### Participant Characteristics

Our analysis included 101,832 individuals, including 52,299 women (51.4%) and 49,533 (48.6%) men. Their overall mean (SD) age was 62.4 (5.3) years. The median follow-up time was 17.0 years, with 24,141 deaths recorded over a total 1,672,715 person-years of follow-up. These deaths included 7,161 from cancer, 7,534 from CVD, and 9,446 from all other causes combined. Baseline characteristics by quintiles of specific dietary fat intake are shown in Table 1. The participants with higher intake of SFAs, PUFAs, or MUFAs tended to be younger and more obese, and were more likely to be male and white, be current smokers or drinkers, use aspirin, have diabetes mellitus, and have a higher intake of fruits and vegetables.

**Table 1:** Characteristics of the participants by quintiles of dietary fat intake in the PLCO study.

**Dietary fat**	**SFAs intake**			**PUFAs intake**			**MUFAs intake**		
**Quintile**	**Q1**	**Q3**	**Q5**	**Q1**	**Q3**	**Q5**	**Q1**	**Q3**	**Q5**
Number of participants	*n* = 20,390	*n* = 20,369	*n* = 20,366	*n* = 20,387	*n* = 20,315	*n* = 20,337	*n* = 20,406	*n* = 20,379	*n* = 20,365
Age (y), mean	63.2	62.4	61.6	63.1	62.4	61.7	63.2	62.4	61.6
Female, %	69.2	53.9	28.1	64.5	52.4	34.9	70.3	53.6	27.4
White, %	85.5	92.3	93.3	89.8	91.9	89.9	88.1	91.8	91.7
BMI, kg/m^2^	26.3	27.2	28.2	26.7	27.2	27.9	26.5	27.2	28.1
Control arm, %	50.7	48.6	48	50.4	49.1	47.7	50.9	49.1	47.4
Total energy, kcal/d, mean	1045.1	1627	2699.5	1046.6	1643.3	2654.1	1028.4	1623	2731
≤ High school, %	41.8	41.1	44.9	44.3	41.3	42.1	43.1	40.9	43.6
Married, %	73.2	79.2	80.5	73.1	79.6	80.3	72.5	79.4	81.3
Regular use of aspirin, %	45.2	47.1	48.9	45	47.1	49.3	44.9	46.7	49.9
Current smokers, %	6.2	8.5	14	8.5	8.7	11.4	6.9	8.7	12.9
Current drinkers, %	68.8	73.8	74.5	69.1	73.5	73.8	68.4	73.9	74.7
History of hypertension, %	33.5	32.8	31.7	33.6	32.2	32.4	33.5	32.2	32.3
History of diabetes, %	6.6	6.6	7.2	6.6	6.6	7.8	6.4	6.5	7.7
Heart disease, %	9.6	8	7.5	9.3	8.3	7.8	9	8	8.1
Stroke, %	2.3	1.8	1.8	2.4	2.1	1.9	2.3	1.9	1.9
Fruit (g/day), mean	267.2	276	280.9	240.3	272.9	307.8	263.1	273.6	286.6
Vegetables (g/day), mean	218.6	276.8	365.6	186	274.1	402	208.2	275.6	381.5

*PLCO, Prostate, Lung, Colorectal and Ovarian; BMI, body mass index; SFAs, saturated fatty acids; PUFAs, polyunsaturated fatty acids; MUFAs, monounsaturated fatty acids; y, year*.

### Dietary Fats and Total Mortality

**Figure 1:**
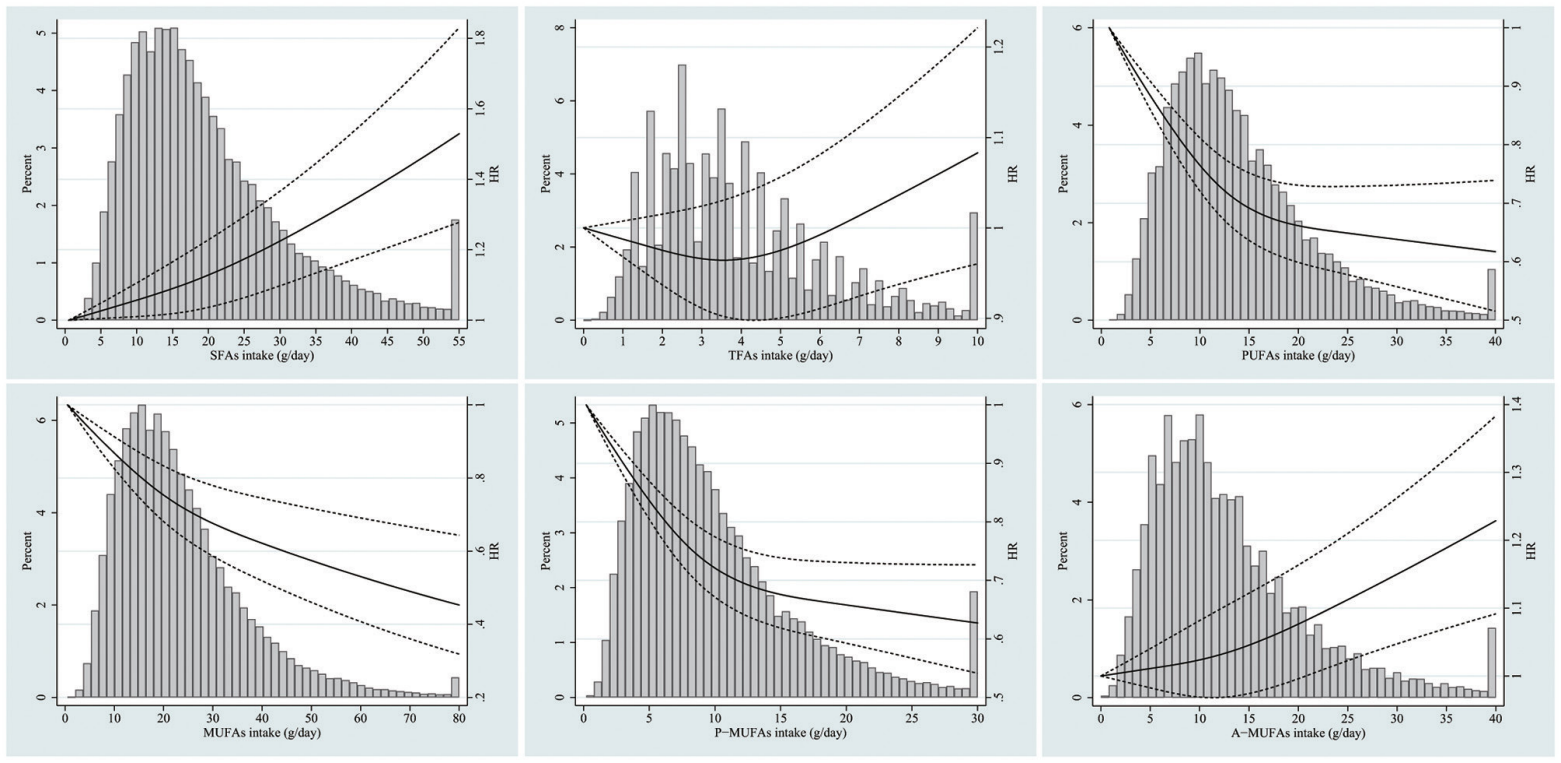
Dose-response analyses for the association between intake of specific dietary fat and total mortality were performed using restricted cubic spline model. Solid lines represent point estimates and dashed lines represent 95% confidence intervals (CIs). Multivariable Hazard ratios (HRs) were calculated by restricted cubic spline regression (using three knots at 10th, 50th, and 90th percentiles) adjusting for age, gender, race, body mass index, education, smoking status, total energy intake, alcohol drinking status, study center, marital status, randomization arm, aspirin use, history of hypertension, history of diabetes, history of stroke, history of heart attack, vegetables intake, fruit intake, and other remaining fatty acids. The histograms show the percentage of participants (left y axis) belonging to each level of specific dietary fat.

Dietary intake of total fat was inversely associated with all-cause mortality in all models (*P*_for trend_ < 0.001). There was a significant positive association between SFA consumption and total mortality (Model 4: HR_*Q*5 *vs*. *Q*1_ = 1.13, 95% CI 1.05–1.22; *P*_for trend_ < 0.001). The corresponding adjusted HR was 1.11 (95% CI 1.07–1.15) per one SD increment of SFAs. No significant association with all-cause mortality was observed for TFA intake (Model 4: HR_*Q*5 *vs*. *Q*1_ = 0.97, 95% CI 0.91–1.04; *P*_for trend_ = 0.899). However, the corresponding adjusted HR was 1.04 (95% CI 1.01–1.07) per one SD increment of TFAs. The PUFA intake was strongly and inversely associated with total mortality in the fully adjusted model (Model 4: HR_*Q*5 *vs*. *Q*1_ = 0.79, 95% CI 0.73–0.85; *P*_for trend_ < 0.001). The corresponding adjusted HR was 0.93 (95% CI 0.90–0.96) per one SD increment of PUFAs. There was a negative association between MUFA intake and total mortality (Model 4: HR_*Q*5 *vs*. *Q*1_ = 0.91, 95% CI 0.84–0.99; *P*_for trend_ = 0.044). The corresponding adjusted HR was 0.89 (95% CI 0.84–0.94) per one SD increment of MUFAs. In terms of MUFAs from different sources, A-MUFA intake was not significantly correlated with total mortality (Model 4: HR_*Q*5 *vs*. *Q*1_ = 1.03, 95% CI 0.96–1.11; *P*_for trend_ = 0.250), whereas P-MUFA intake was inversely associated with total mortality (Model 4: HR_*Q*5 *vs*. *Q*1_ = 0.83, 95% CI 0.77–0.89; *P*_for trend_ < 0.001). The corresponding adjusted HR was 0.93 (95% CI 0.90–0.96) per 1 SD increment of P-MUFAs (Table 2). Spline regression plots of total mortality in relation to the intake of specific dietary fat are shown in Figure 1.

**Table 2:** Associations of total and specific dietary fat intake with all-cause mortality.

**Variables (g/day)**	**Median**	**Cohort (*n*)**	**Cases (*n*)**	**Model 1^a^**	**Model 2^b^**	**Model 3^c^**	**Model 4^d^**
**Total fat**							
Q1 (<35.30)	27.61	20,382	5,007	Reference group	Reference group	Reference group	
Q2 (≥35.30 to <48.48)	41.95	20,366	4,594	0.94 (0.90–0.98), *p* = 0.002	0.92 (0.89–0.96), *p* < 0.001	0.93 (0.89–0.97), *p* = 0.001	
Q3 (≥48.48 to <63.09)	55.33	20,362	4,652	0.94 (0.90–0.98), *p* = 0.002	0.90 (0.86–0.94), *p* < 0.001	0.91 (0.87–0.95), *p* < 0.001	
Q4 (≥63.09 to <85.23)	72.38	20,362	4,772	0.95 (0.92–0.99), *p* = 0.024	0.88 (0.83–0.91), *p* < 0.001	0.89 (0.84–0.93), *p* < 0.001	
Q5 (≥85.23)	106.11	20,365	5,116	1.05 (1.01–1.10), *p* = 0.013	0.85 (0.79–0.90), *p* < 0.001	0.88 (0.83–0.94), *p* < 0.001	
				*p*_for trend_ <0.001	*p*_for trend_ <0.001	*p*_for trend_ <0.001	
**SFAs**							
Q1 (<10.62)	8.23	20,390	4,861	Reference group	Reference group	Reference group	Reference group
Q2 (≥10.62 to <14.93)	12.76	20,373	4,554	0.96 (0.92–1.00), *p* = 0.054	0.94 (0.90–0.98), *p* = 0.004	0.95 (0.91–0.99), *p* = 0.022	0.97 (0.93–1.02), *p* = 0.215
Q3 (≥ 14.93 to <19.86)	17.21	20,369	4,621	0.98 (0.94–1.02), *p* = 0.235	0.94 (0.90–0.98), *p* = 0.005	0.96 (0.92–1.01), *p* = 0.089	1.00 (0.96–1.05), *p* = 0.886
Q4 (≥ 19.86 to <27.53)	23.07	20,339	4,823	1.02 (0.98–1.06), *p* = 0.34	0.94 (0.90–0.99), *p* = 0.017	0.97 (0.92–1.02), *p* = 0.192	1.03 (0.98–1.09), *p* = 0.287
Q5 (≥ 27.53)	34.96	20,366	5,282	1.14 (1.09–1.19), *p* < 0.001	0.97 (0.91–1.03), *p* = 0.320	1.02 (0.96–1.08), *p* = 0.586	1.13 (1.05–1.22), *p* = 0.001
				*p*_for trend_ <0.001	*p*_for trend_ = 0.805	*p*_for trend_ = 0.215	*p*_for trend_ <0.001
**TFAs**							
Q1 (<2.08)	1.57	20,523	4,870	Reference group	Reference group	Reference group	Reference group
Q2 (≥ 2.08 to <2.98)	2.53	20,366	4,482	0.95 (0.91–0.99), *p* = 0.007	0.92 (0.88–0.96), *p* < 0.001	0.92 (0.88–0.96), *p* < 0.001	0.93 (0.89–0.97), *p* = 0.001
Q3 (≥ 2.98 to <4.00)	3.45	20,291	4,637	0.98 (0.94–1.02), *p* = 0.239	0.93 (0.89–0.97), *p* = 0.001	0.93 (0.89–0.97), *p* = 0.002	0.95 (0.91–0.99), *p* = 0.028
Q4 (≥ 4.00 to <5.58)	4.67	20,343	4,967	1.03 (0.99–1.07), *p* = 0.147	0.95 (0.90–0.99), *p* = 0.019	0.95 (0.91–1.00), *p* = 0.052	0.98 (0.93–1.03), *p* = 0.475
Q5 (≥ 5.58)	7.09	20,314	5,185	1.09 (1.05–1.13), *p* < 0.001	0.91 (0.86–0.96), *p* = 0.001	0.92 (0.87–0.98), *p* = 0.005	0.97 (0.91–1.04), *p* = 0.372
				*p*_for trend_ <0.001	*p*_for trend_ = 0.036	*p*_for trend_ = 0.091	*p*_for trend_ = 0.899
**PUFAs**							
Q1 (<7.95)	6.21	20,387	5,298	Reference group	Reference group	Reference group	Reference group
Q2 (≥ 7.95 to <10.93)	9.47	20,409	4,748	0.90 (0.87–0.94), *p* < 0.001	0.89 (0.86–0.93), *p* < 0.001	0.90 (0.86–0.93), *p* < 0.001	0.90 (0.87–0.94), *p* < 0.001
Q3 (≥ 10.93 to <14.26)	12.49	20,315	4,641	0.87 (0.84–0.91), *p* < 0.001	0.83 (0.80–0.87), *p* < 0.001	0.84 (0.80–0.87), *p* < 0.001	0.85 (0.81–0.89), *p* < 0.001
Q4 (≥ 14.26 to <19.21)	16.37	20,389	4,644	0.88 (0.84–0.91), *p* < 0.001	0.78 (0.75–0.82), *p* < 0.001	0.80 (0.76–0.83), *p* < 0.001	0.82 (0.77–0.86), *p* < 0.001
Q5 (≥ 19.21)	23.89	20,337	4,810	0.92 (0.88–0.96), *p* < 0.001	0.73 (0.69–0.77), *p* < 0.001	0.75 (0.70–0.79), *p* < 0.001	0.79 (0.73–0.85), *p* < 0.001
				*p*_for trend_ = 0.001.	*p*_for trend_ <0.001.	*p*_for trend_ <0.001.	*p*_for trend_ <0.001.
**MUFAs**							
Q1 (<12.95)	9.96	20,406	5,022	Reference group	Reference group	Reference group	Reference group
Q2 (≥ 12.95 to <18.07)	15.52	20,343	4,564	0.93 (0.89–0.97), *p* < 0.001	0.91 (0.87–0.95), *p* < 0.001	0.92 (0.88–0.96), *p* < 0.001	0.93 (0.89–0.97), *p* = 0.001
Q3 (≥ 18.07 to <23.80)	20.77	20,379	4,605	0.92 (0.89–0.96), *p* < 0.001	0.88 (0.84–0.92), *p* < 0.001	0.89 (0.85–0.93), *p* < 0.001	0.91 (0.87–0.96), *p* < 0.001
Q4 (≥ 23.80 to <32.49)	27.46	20,344	4,843	0.95 (0.92–0.99), *p* = 0.019	0.86 (0.82–0.91), *p* < 0.001	0.88 (0.84–0.92), *p* < 0.001	0.91 (0.86–0.96), *p* = 0.001
Q5 (≥ 32.49)	40.66	20,365	5,107	1.04 (1.00–1.08), *p* = 0.081	0.83 (0.77–0.88), *p* < 0.001	0.85 (0.80–0.91), *p* < 0.001	0.91 (0.84–0.99), *p* = 0.021
				*p*_for trend_ = 0.001	*p*_for trend_ <0.001	*p*_for trend_ <0.001	*p*_for trend_ = 0.044
**P-MUFAs**							
Q1 (<4.88)	3.65	20,414	5260	Reference group	Reference group	Reference group	Reference group
Q2 (≥ 4.88 to <7.18)	6.01	20,340	4840	0.92 (0.88–0.95), *p* < 0.001	0.92 (0.89–0.96), *p* < 0.001	0.93 (0.89–0.97), *p* < 0.001	0.94 (0.90–0.98), *p* = 0.003
Q3 (≥ 7.18 to <9.89 )	8.44	20,393	4611	0.87 (0.83–0.90), *p* < 0.001	0.85 (0.81–0.89), *p* < 0.001	0.85 (0.82–0.89), *p* < 0.001	0.87 (0.83–0.91), *p* < 0.001
Q4 (≥ 9.89 to <14.16 )	11.67	20,355	4654	0.85 (0.82–0.89), *p* < 0.001	0.80 (0.77–0.84), *p* < 0.001	0.81 (0.77–0.85), *p* < 0.001	0.84 (0.80–0.89), *p* < 0.001
Q5 (≥ 14.16)	18.44	20,335	4776	0.87 (0.84–0.91), *p* < 0.001	0.76 (0.72–0.80), *p* < 0.001	0.77 (0.73–0.81), *p* < 0.001	0.83 (0.77–0.89), *p* < 0.001
				*p*_for trend_ <0.001	*p*_for trend_ <0.001	*p*_for trend_ <0.001	*p*_for trend_ <0.001
**A-MUFAs**							
Q1 (<6.63 )	4.95	20,388	4702	Reference group	Reference group	Reference group	Reference group
Q2 (≥ 6.63 to <9.64)	8.11	20,393	4620	1.02 (0.98–1.06), *p* = 0.332	0.99 (0.95–1.04), *p* = 0.773	1.00 (0.96–1.05), *p* = 0.862	1.00 (0.96–1.04), *p* = 0.998
Q3 (≥ 9.64 to <13.08 )	11.23	20,338	4484	0.98 (0.94–1.02), *p* = 0.425	0.94 (0.90–0.99), *p* = 0.013	0.96 (0.92–1.01), *p* = 0.110	0.96 (0.91–1.00), *p* = 0.061
Q4 (≥ 13.08 to <18.48)	15.33	20,358	5002	1.09 (1.05–1.14), *p* < 0.001	1.01 (0.97–1.07), *p* = 0.472	1.04 (0.99–1.09), *p* = 0.086	1.02 (0.97–1.08), *p* = 0.39
Q5 (≥18.48 )	23.76	20,360	5333	1.20 (1.15–1.25), *p* < 0.001	1.04 (0.98–1.10), *p* = 0.213	1.08 (1.02–1.15), *p* = 0.009	1.03 (0.96–1.11), *p* = 0.402
				*p*_for trend_ <0.001	*p*_for trend_ = 0.065	*p*_for trend_ = 0.001	*p*_for trend_ = 0.250

*SFAs, saturated fatty acids; PUFAs, polyunsaturated fatty acids; MUFAs, monounsaturated fatty acids; TFAs, trans-fatty acids; P-MUFAs, MUFAs from plant; A-MUFAs, MUFAs from animal; Q, quintile*.

a *Adjusted for age (continuous) and gender (male vs. female)*.

b *Additionally adjusted for race (non-Hispanic White vs. Other), body mass index (continuous), education (≤ high school vs. ≥ some college), smoking status (never vs. former ≤ 15 years since quit vs. former > 15 years since quit vs. former year since quit unknown vs. current smoker ≤ 1 pack per day vs. current smoker > 1 pack per day vs. current smoker intensity unknown), total energy intake (continuous), alcohol drinking status (never vs. former vs. current), study center (categorical), marital status (married vs. not married), randomization arm (screening group vs. control group), aspirin use (yes vs. no), history of hypertension (yes vs. no), history of diabetes (yes vs. no), vegetables intake (continuous), and fruit intake (continuous)*.

c *Further adjusted for history of stroke (yes vs. no) and history of heart attack (yes vs. no)*.

d *Additionally adjusted for other remaining fatty acids*.

### Dietary Fats and Cause-Specific Mortality

Dietary intake of SFAs or TFAs was not associated with CVD mortality either in age- and gender-adjusted analyses (*P*_for trend_ > 0.05) or in the fully adjusted model (*P*_for trend_ > 0.05) (Supplementary Table 1). There was a significant inverse association between total fat intake and CVD mortality (HR_*Q*5 *vs*. *Q*1_ = 0.81, 95% CI 0.72–0.91; *P*_for trend_ = 0.001). The consumption of PUFAs (HR_*Q*5 *vs*. *Q*1_ = 0.66, 95% CI 0.58–0.75; *P*_for trend_ < 0.001) and MUFAs (HR_*Q*5 *vs*. *Q*1_ = 0.85, 95% CI 0.73–0.98; *P*_for trend_ = 0.020) was negatively associated with CVD mortality in the fully adjusted model. In terms of MUFAs from different sources, P-MUFA intake was significantly inversely correlated with CVD mortality (Model 4: HR_*Q*5 *vs*. *Q*1_ = 0.83, 95% CI 0.73–0.94; *P*_for trend_ = 0.004), whereas A-MUFA intake was not significantly associated with CVD mortality (Model 4: HR_*Q*5 *vs*. *Q*1_ = 1.10, 95% CI 0.96–1.26; *P*_for trend_ = 0.215). Dietary intake of total fat, SFAs, TFAs, MUFAs, and A-MUFAs were significantly associated with cancer mortality in age- and gender-adjusted analyses (Supplementary Table 2, *P*_for trend_ < 0.05). However, these associations were not significant in the fully adjusted Model 4 (*P*_for trend_ > 0.05). There was no significant association between intake of PUFAs or P-MUFAs and cancer mortality in all models. Spline regression plots of CVD or cancer mortality in relation to intake of specific dietary fat are shown in Supplementary Figures 1, 2.

### Sensitivity Analyses and Subgroup Analyses

The significant associations of specific dietary fats, including total fat, SFAs, PUFAs, MUFAs, and P-MUFAs, with total mortality made little change after excluding the first 5 years of follow-up. We further performed a sensitivity analysis using ratio of fat intake to total energy intake as exposure, and similar results were obtained. The results of subgroup analyses for the associations between specific dietary fats and total mortality based on gender, age, BMI, arm, education, drinking status, smoking status, and married status are summarized in Table 3.

**Table 3:** Subgroup analyses of the associations of total and specific dietary fat intake with total mortality.

	**Total fat**	**SFAs**	**TFAs**	**PUFAs**	**MUFAs**	**P-MUFAs**	**A-MUFAs**
**Gender**							
Male	0.94 (0.91–0.97), *p* < 0.001	1.09 (1.04–1.14), *p* < 0.001	1.08 (1.02–1.13), *p* = 0.006	0.92 (0.88–0.96), *p* < 0.001	0.91 (0.85–0.98), *p* = 0.010	0.94 (0.91–0.98), *p* = 0.001	1.03 (0.97–1.09), *p* = 0.381
Female	0.97 (0.91–1.03), *p* = 0.339	1.20 (1.12–1.30), *p* < 0.001	1.03 (1.00–1.07), *p* = 0.041	0.97 (0.91–1.03), *p* = 0.302	0.81 (0.72–0.91), *p* = 0.001	0.91 (0.85–0.97), *p* = 0.002	0.93 (0.84–1.03), *p* = 0.156
**Age (y)**							
<65	0.94 (0.91–0.98), *p* = 0.002	1.10 (1.04–1.16), *p* = 0.001	1.02 (0.98–1.06), *p* = 0.383	0.91 (0.86–0.95), *p* < 0.001	0.93 (0.85–1.01), *p* = 0.091	0.96 (0.92–1.00), *p* = 0.070	1.07 (0.99–1.14), *p* = 0.070
≥ 65	0.94 (0.90–0.98), *p* = 0.003	1.13 (1.07–1.19), *p* < 0.001	1.09 (1.05–1.13), *p* < 0.001	0.96 (0.91–1.01), *p* = 0.089	0.82 (0.76–0.90), *p* < 0.001	0.91 (0.87–0.95), *p* < 0.001	0.95 (0.88–1.02), *p* = 0.147
**BMI (kg/m** ^**2**^ **)**							
<25.0	0.95 (0.90–1.00), *p* = 0.046	1.16 (1.09–1.24), *p* < 0.001	1.04 (0.99–1.09), *p* = 0.104	0.92 (0.87–0.98), *p* = 0.011	0.87 (0.78–0.96), *p* = 0.008	0.93 (0.88–0.98), *p* = 0.012	0.97 (0.89–1.07), *p* = 0.576
≥ 25.0	0.96 (0.93–0.99), *p* = 0.008	1.10 (1.05–1.15), *p* < 0.001	1.05 (1.01–1.08), *p* = 0.007	0.93 (0.89–0.97), *p* < 0.001	0.91 (0.85–0.98), *p* = 0.009	0.93 (0.90–0.97), *p* < 0.001	1.05 (0.99–1.11), *p* = 0.111
**Arm**							
Intervention	0.94 (0.91–0.98), *p* = 0.003	1.14 (1.08–1.20), *p* < 0.001	1.08 (1.04–1.12), *p* < 0.001	0.91 (0.87–0.96), *p* < 0.001	0.86 (0.79–0.94), *p* = 0.001	0.93 (0.89–0.97), *p* = 0.001	0.96 (0.90–1.03), p = 0.313
Control	0.94 (0.90–0.98), *p* = 0.001	1.08 (1.03–1.14), *p* = 0.003	1.01 (0.97–1.05), *p* = 0.780	0.94 (0.89–0.99), *p* = 0.013	0.92 (0.84–1.00), *p* = 0.046	0.94 (0.90–0.98), *p* = 0.009	1.05 (0.98–1.13), *p* = 0.148
**Education**							
≤ High school	0.96 (0.92–0.99), *p* = 0.025	1.11 (1.05–1.17), *p* < 0.001	1.06 (1.02–1.11), *p* = 0.002	0.95 (0.90–1.00), *p* = 0.055	0.86 (0.79–0.94), *p* = 0.001	0.91 (0.87–0.96), *p* < 0.001	0.96 (0.90–1.03), *p* = 0.285
≥ Some college	0.93 (0.90–0.97), *p* < 0.001	1.11 (1.06–1.17), *p* < 0.001	1.02 (0.98–1.06), *p* = 0.259	0.91 (0.87–0.95), *p* < 0.001	0.91 (0.84–0.98), *p* = 0.017	0.95 (0.91–0.99), *p* = 0.009	1.05 (0.98–1.13), *p* = 0.151
**Drinking status**							
Never	0.98 (0.86–1.11), *p* = 0.717	1.16 (1.01–1.34), *p* = 0.04	1.08 (0.98–1.19), *p* = 0.121	0.98 (0.87–1.11), *p* = 0.770	0.84 (0.67–1.05), *p* = 0.124	0.96 (0.85–1.07), *p* = 0.454	0.83 (0.69–1.00), *p* = 0.045
Former	0.93 (0.86–1.00), *p* = 0.064	1.06 (0.97–1.16), *p* = 0.175	1.04 (0.98–1.10), *p* = 0.228	0.92 (0.85–0.99), *p* = 0.034	0.94 (0.82–1.07), *p* = 0.326	0.93 (0.87–1.00), *p* = 0.051	1.15 (1.03–1.28), *p* = 0.012
Current	0.95 (0.92–0.98), *p* < 0.001	1.12 (1.07–1.17), *p* < 0.001	1.04 (1.01–1.08), *p* = 0.012	0.92 (0.89–0.96), *p* < 0.001	0.88 (0.82–0.95), *p* = 0.001	0.93 (0.90–0.97), *p* < 0.001	0.99 (0.93–1.05), *p* = 0.802
**Smoking status**							
Never	0.97 (0.92–1.03), *p* = 0.347	1.12 (1.05–1.20), *p* = 0.001	1.05 (1.00–1.11), *p* = 0.032	0.91 (0.86–0.97), *p* = 0.002	0.93 (0.84–1.03), *p* = 0.175	0.96 (0.91–1.01), *p* = 0.152	1.02 (0.93–1.11), *p* = 0.697
Current	0.93 (0.87–0.98), *p* = 0.007	1.04 (0.96–1.13), *p* = 0.322	1.03 (0.97–1.10), *p* = 0.349	0.90 (0.82–0.98), *p* = 0.012	0.95 (0.82–1.10), *p* = 0.482	0.99 (0.91–1.06), *p* = 0.705	0.99 (0.89–1.10), *p* = 0.859
Former	0.95 (0.91–0.99), *p* = 0.007	1.16 (1.10–1.22), *p* < 0.001	1.04 (1.00–1.08), *p* = 0.05	0.95 (0.91–1.00), *p* = 0.045	0.84 (0.78–0.92), *p* < 0.001	0.90 (0.86–0.94), *p* < 0.001	1.02 (0.95–1.10), *p* = 0.516
**Married status**							
Not married	0.94 (0.91–0.97), *p* < 0.001	1.10 (1.05–1.14), *p* < 0.001	1.03 (1.00–1.07), *p* = 0.041	0.93 (0.89–0.97), *p* < 0.001	0.90 (0.84–0.97), *p* = 0.004	0.94 (0.90–0.97), *p* < 0.001	1.03 (0.97–1.09), *p* = 0.360
Married	0.96 (0.90–1.01), *p* = 0.113	1.17 (1.09–1.26), *p* < 0.001	1.07 (1.02–1.13), *p* = 0.011	0.94 (0.88–1.00), *p* = 0.056	0.83 (0.74–0.94), *p* = 0.002	0.91 (0.86–0.97), *p* = 0.005	0.94 (0.85–1.03), *p* = 0.178

*SFAs, saturated fatty acids; PUFAs, polyunsaturated fatty acids; MUFAs, monounsaturated fatty acids; TFAs, trans-fatty acids; P-MUFAs, MUFAs from plant; A-MUFAs, MUFAs from animal; y, year; BMI, body mass index*.

*HRs were for per one SD increment of specific dietary fats and were adjusted for age (continuous), gender (male vs. female), race (non-Hispanic White vs. Other), body mass index (continuous), education (≤ high school vs. ≥ some college), smoking status (never vs. former ≤ 15 years since quit vs. former > 15 years since quit vs. former year since quit unknown vs. current smoker ≤ 1 pack per day vs. current smoker > 1 pack per day vs. current smoker intensity unknown), total energy intake (continuous), alcohol drinking status (never vs. former vs. current), study center (categorical), marital status (married vs. not married), randomization arm (screening group vs. control group), aspirin use (yes vs. no), history of hypertension (yes vs. no), history of diabetes (yes vs. no), history of stroke (yes vs. no), history of heart attack (yes vs. no), vegetables intake (continuous), fruit intake (continuous), and other remaining fatty acids*.

## Discussion

This large prospective cohort study found that participants with higher intake of PUFAs or P-MUFAs had a lower incidence of all-cause death and CVD mortality, whereas those with higher intake of SFAs had a greater risk of total mortality. All types of dietary fats were not associated with cancer mortality.

Effects of reducing SFA intake by replacing SFA with other nutrients have been systematically reviewed. Replacement of SFA with PUFA, MUFA, or carbohydrates can cause a significant decrease in cholesterol (18). Our positive findings of SFA intake in relation to total mortality was concordant with a recent prospective study based on NHANES cohort by Mazidi et al. (4), which reported that SFA intake was significantly associated with a higher risk of total mortality (HR: 1.08, 95% CI: 1.04–1.11). However, Mazidi et al. (4) also performed a meta-analysis of 18 prospective studies and only observed a non-significant association between SFA intake and total mortality (HR: 1.04, 95% CI: 0.98–1.11) with obvious heterogeneity across the included studies. This was because previous studies reported positive, negative, or null results between SFA intake and all-cause mortality. Therefore, although the U.S. Dietary Guidelines recommend the restriction of SFA intake to <10% of calories, there is no robust evidence that current population-wide arbitrary upper limits on SFA consumption in the United States will reduce mortality (19).

Previous data on the association between MUFA intake and mortality have been inconsistent. A recent meta-analysis of prospective cohort studies found that MUFA intake was associated with 7% lower risk of all-cause mortality and 20% lower risk of stroke mortality (4). However, substantial between-study heterogeneity was observed, partly because of the inconsistent adjustment of variables across individual studies. Another possible reason could be that MUFAs have diverse food sources. Guasch-Ferré et al. (8) reported that MUFAs from plant and animal sources had different associations with total and cause-specific mortality. The adjusted HRs (95% CIs) for participants in the highest quintile of P-MUFAs and A-MUFAs, as compared with those in the lowest quintile, were 0.84 (0.80–0.89; *P*_for trend_ <0.001) and 1.16 (1.08–1.24; *P*_for trend_ < 0.001), respectively. The results of our study indicated that higher intake of P-MUFAs was associated with a lower risk of death from any or a cardiovascular cause, whereas A-MUFA intake had a positive, *albeit* not significant, relationship with total mortality and CVD. Collectively, the present and previous studies indicate the importance and diverse effects of primary dietary MUFA sources.

Our observation of a strong inverse association between PUFA intake and mortality does not stand alone in the literature. PUFAs have been consistently inversely associated with total mortality and cause-specific mortality in observational studies (20, 21). Recently, a meta-analysis based on 29 prospective cohorts with 1,148,117 participants found that a greater consumption of PUFAs was associated with lower risks of total mortality and stroke mortality (4). PUFAs, especially omega-3 PUFAs, have been shown to favorably reduce the risk factors of cardiovascular disease (22). A recent meta-analysis evaluated the effect of omega-3 dosage on cardiovascular outcomes based on interventional trials and found that omega-3 supplementation was an effective preventive strategy for CVD and the protective effect appeared to be linearly related to dosage (23). Considering benefits likely outweigh the risks, the American Heart Association report offered a Class IIa recommendation for the use of omega-3 PUFA supplements (24).

Replacement of TFA with PUFA or MUFA can significantly reduce total cholesterol, low-density lipoprotein cholesterol, and triglycerides (25). Evidence on the association between TFA intake and mortality was relatively limited. An early meta-analysis based on only two prospective studies reported that total TFA intake was positively associated with all-cause mortality (HR 1.34, 95% CI 1.16–1.56) (2). Several recent prospective studies based on NIH–AARP Diet and Health Study, Nurses' Health Study, and Health Professionals Follow-up Study also found that a higher consumption of TFAs was associated with a higher risk of mortality (9, 26). Because of the potential adverse effects of TFAs, several countries have implemented policies to reduce industrial TFA (iTFA) consumption. Rubinstein et al. (27) and Marklund et al. (28) reported that elimination of iTFA can cost-effectively improve health equality in Australia and in Argentina, respectively. In our study, we did not find a significant association between TFA intake, as a categorical variable, and all-cause mortality; nevertheless, when TFA intake was treated as a continuous variable, the association turned out to be significant, with a fully adjusted HR of 1.04 (95% CI 1.01–1.07) per 1 SD increment of TFAs.

The strengths of the current study included the prospective design, a large population size (24,141 deaths among 101,832 participants), and a long duration of the 17-year follow-up, which substantially decreased the chance of reverse causality and provided a robust power to examine moderate associations between dietary fat intake and mortality risk. The large number of deaths enabled a robust examination of cause-specific mortality. The broad ranges of dietary fat intake allowed us to comprehensively evaluate the effects of dietary fat at diverse intake levels.

This study had several limitations. First, despite full adjustment for established and suspected confounders, we could not exclude the possibility of residual or unmeasured confounding. For example, physical activity information was not available and we could not exclude under-reporters of energy intake. Second, the causality could not be established due to the observational study setting. Furthermore, the observed associations were possibly interpreted by specific food or dietary patterns. For example, sources of MUFA include a wide variety of foods and MUFA is also a part of various dietary patterns. Third, inherent measurement errors (e.g., underreporting of dietary intake) may be still present in the analyses, which would underestimate the true measure of effect, as a result of non-differential misclassification and biasing risk estimates toward the null. Fourth, most of participants included in this study were non-Hispanic Whites, which may limit its generalizability to other populations. Finally, only a single measurement for dietary intake was performed at baseline and it was possible that participant diets may have changed over time.

In conclusion, this study observed the detrimental effects of SFA intake on total mortality. A higher intake of PUFAs or MUFAs, especially P-MUFAs, was associated with lower risks of death from any cause or cardiovascular cause. Overall, these data support current dietary recommendations to replace SFAs with PUFAs and P-MUFAs for the prevention of chronic diseases and premature deaths.

## Data Availability Statement

Publicly available datasets were analyzed in this study. This data can be found here: https://cdas.cancer.gov/datasets/plco/.

## Ethics Statement

The studies involving human participants were reviewed and approved by National Cancer Institute. The patients/participants provided their written informed consent to participate in this study.

## Author Contributions

XY and XX contributed to the conception or design of the work and drafted the manuscript. XY, XX, SW, and DX contributed to the acquisition, analysis, or interpretation of data for the work. SW and DX critically revised the manuscript. All authors gave final approval and agree to be accountable for all aspects of work ensuring integrity and accuracy.

## Author Disclaimer

The statements contained herein are solely those of the authors and do not represent or imply concurrence or endorsement by NCI.

## Conflict of Interest

The authors declare that the research was conducted in the absence of any commercial or financial relationships that could be construed as a potential conflict of interest.

## Publisher's Note

All claims expressed in this article are solely those of the authors and do not necessarily represent those of their affiliated organizations, or those of the publisher, the editors and the reviewers. Any product that may be evaluated in this article, or claim that may be made by its manufacturer, is not guaranteed or endorsed by the publisher.

## Supplementary Material

Supplementary Figure 1 Dose-response analyses for the association between intake of specific dietary fat and cardiovascular disease (CVD) mortality were performed using restricted cubic spline model. Solid lines represent point estimates and dashed lines represent 95% confidence intervals (CIs). Multivariable Hazard ratios (HRs) were calculated by restricted cubic spline regression (using three knots at 10th, 50th, and 90th percentiles) adjusting for age, gender, race, body mass index, education, smoking status, total energy intake, alcohol drinking status, study center, marital status, randomization arm, aspirin use, history of hypertension, history of diabetes, history of stroke, history of heart attack, vegetables intake, fruit intake, and other remaining fatty acids. The histograms show the percentage of participants (left y axis) belonging to each level of specific dietary fat.

Supplementary Figure 2 Dose-response analyses for the association between intake of specific dietary fat and cancer mortality were performed using restricted cubic spline model. Solid lines represent point estimates and dashed lines represent 95% confidence intervals (CIs). Multivariable Hazard ratios (HRs) were calculated by restricted cubic spline regression (using three knots at 10th, 50th, and 90th percentiles) adjusting for age, gender, race, body mass index, education, smoking status, total energy intake, alcohol drinking status, study center, marital status, randomization arm, aspirin use, history of hypertension, history of diabetes, history of stroke, history of heart attack, vegetables intake, fruit intake, and other remaining fatty acids. The histograms show the percentage of participants (left y axis) belonging to each level of specific dietary fat.
