# Supplementary material for: Associations of Dietary Fat Intake With Mortality From All Causes, Cardiovascular Disease, and Cancer: A Prospective Study
Source: Front Nutr. 2021 Aug 9;8:701430. doi: 10.3389/fnut.2021.701430 (PMC8380819; doi:10.3389/fnut.2021.701430)
Supplement: Supplementary file 2 [file Table_2.DOCX]

**Supplemental Table S2 Associations of total and specific dietary fat intake with cancer mortality**

| Variables (g/day) | Median | Cohort (n) | Cases (n) | Model 1^a^ | Model 2^b^ | Model 3^c^ | Model 4^d^ |
| --- | --- | --- | --- | --- | --- | --- | --- |
| Total fat |  |  |  |  |  |  |  |
| Q1 (< 35.30) | 27.61 | 20,382 | 1,362 | Reference group | Reference group | Reference group |  |
| Q2 (≥ 35.30 to < 48.48) | 41.95 | 20,366 | 1,326 | 0.98 (0.91-1.05), p=0.539 | 0.96 (0.89-1.04), p=0.311 | 0.96 (0.89-1.04), p=0.347 |  |
| Q3 (≥ 48.48 to < 63.09) | 55.33 | 20,362 | 1,385 | 1.00 (0.93-1.08), p=0.989 | 0.93 (0.86-1.01), p=0.095 | 0.94 (0.87-1.02), p=0.141 |  |
| Q4 (≥ 63.09 to < 85.23) | 72.38 | 20,362 | 1,473 | 1.05 (0.97-1.13), p=0.232 | 0.93 (0.85-1.02), p=0.124 | 0.94 (0.86-1.03), p=0.188 |  |
| Q5 (≥ 85.23) | 106.11 | 20,365 | 1,615 | 1.15 (1.06-1.24), p<0.001 | 0.90 (0.80-1.02), p=0.094 | 0.92 (0.82-1.03), p=0.155 |  |
|  |  |  |  | p _for trend_<0.001 | p _for trend_=0.119 | p _for trend_=0.196 |  |
| SFAs |  |  |  |  |  |  |  |
| Q1 (< 10.62) | 8.23 | 20,390 | 1,322 | Reference group | Reference group | Reference group | Reference group |
| Q2 (≥ 10.62 to < 14.93 ) | 12.76 | 20,373 | 1,357 | 1.03 (0.96-1.11), p=0.412 | 0.99 (0.92-1.07), p=0.860 | 1.00 (0.92-1.08), p=0.968 | 1.01 (0.94-1.10), p=0.741 |
| Q3 (≥ 14.93 to < 19.86 ) | 17.21 | 20,369 | 1,378 | 1.04 (0.96-1.12), p=0.313 | 0.97 (0.89-1.05), p=0.482 | 0.98 (0.90-1.06), p=0.637 | 1.01 (0.93-1.10), p=0.834 |
| Q4 (≥ 19.86 to < 27.53) | 23.07 | 20,339 | 1,405 | 1.05 (0.97-1.13), p=0.225 | 0.93 (0.85-1.01), p=0.094 | 0.94 (0.85-1.02), p=0.149 | 0.98 (0.89-1.08), p=0.653 |
| Q5 (≥ 27.53) | 34.96 | 20,366 | 1,699 | 1.27 (1.17-1.36), p<0.001 | 1.01 (0.90-1.13), p=0.842 | 1.03 (0.92-1.15), p=0.623 | 1.12 (0.98-1.27), p=0.108 |
|  |  |  |  | p _for trend_<0.001 | p _for trend_=0.940 | p _for trend_=0.723 | p _for trend_=0.151 |
| TFAs |  |  |  |  |  |  |  |
| Q1 (< 2.08 ) | 1.57 | 20,523 | 1,339 | Reference group | Reference group | Reference group | Reference group |
| Q2 (≥ 2.08 to < 2.98 ) | 2.53 | 20,366 | 1,289 | 0.97 (0.90-1.05), p=0.441 | 0.93 (0.86-1.00), p=0.064 | 0.93 (0.86-1.01), p=0.071 | 0.94 (0.86-1.01), p=0.105 |
| Q3 (≥ 2.98 to < 4.00) | 3.45 | 20,291 | 1,391 | 1.04 (0.96-1.12), p=0.357 | 0.97 (0.89-1.05), p=0.402 | 0.97 (0.89-1.05), p=0.474 | 0.98 (0.91-1.07), p=0.699 |
| Q4 (≥ 4.00 to < 5.58 ) | 4.67 | 20,343 | 1,532 | 1.12 (1.04-1.21), p=0.003 | 0.98 (0.90-1.07), p=0.653 | 0.99 (0.9-1.08), p=0.757 | 1.01 (0.92-1.10), p=0.901 |
| Q5 (≥ 5.58) | 7.09 | 20,314 | 1,610 | 1.17 (1.08-1.26), p<0.001 | 0.93 (0.84-1.04), p=0.186 | 0.94 (0.84-1.04), p=0.226 | 0.97 (0.86-1.09), p=0.64 |
|  |  |  |  | p _for trend_<0.001 | p _for trend_=0.446 | p _for trend_=0.518 | p _for trend_=0.935 |
| PUFAs |  |  |  |  |  |  |  |
| Q1 (< 7.95 ) | 6.21 | 20,387 | 1,422 | Reference group | Reference group | Reference group | Reference group |
| Q2 (≥ 7.95 to < 10.93) | 9.47 | 20,409 | 1,361 | 0.95 (0.88-1.02), p=0.153 | 0.94 (0.87-1.01), p=0.104 | 0.94 (0.87-1.02), p=0.125 | 0.95 (0.88-1.03), p=0.197 |
| Q3 (≥ 10.93 to < 14.26 ) | 12.49 | 20,315 | 1,406 | 0.97 (0.90-1.04), p=0.348 | 0.92 (0.85-1.00), p=0.046 | 0.92 (0.85-1.00), p=0.052 | 0.94 (0.87-1.02), p=0.150 |
| Q4 (≥ 14.26 to < 19.21 ) | 16.37 | 20,389 | 1,445 | 0.98 (0.91-1.06), p=0.637 | 0.89 (0.82-0.97), p=0.009 | 0.90 (0.82-0.98), p=0.015 | 0.92 (0.84-1.02), p=0.103 |
| Q5 (≥ 19.21 ) | 23.89 | 20,337 | 1,527 | 1.03 (0.96-1.11), p=0.398 | 0.86 (0.77-0.96), p=0.006 | 0.87 (0.78-0.97), p=0.010 | 0.92 (0.81-1.05), p=0.221 |
|  |  |  |  | p _for trend_=0.125 | p _for trend_=0.008 | p _for trend_=0.013 | p _for trend_=0.258 |
| MUFAs |  |  |  |  |  |  |  |
| Q1 (< 12.95) | 9.96 | 20,406 | 1,365 | Reference group | Reference group | Reference group | Reference group |
| Q2 (≥ 12.95 to < 18.07) | 15.52 | 20,343 | 1,307 | 0.96 (0.89-1.03), p=0.281 | 0.94 (0.87-1.02), p=0.136 | 0.95 (0.87-1.02), p=0.166 | 0.96 (0.88-1.04), p=0.264 |
| Q3 (≥ 18.07 to < 23.80) | 20.77 | 20,379 | 1,370 | 0.98 (0.91-1.06), p=0.674 | 0.92 (0.84-1.00), p=0.038 | 0.92 (0.85-1.00), p=0.061 | 0.94 (0.86-1.03), p=0.175 |
| Q4 (≥ 23.80 to < 32.49) | 27.46 | 20,344 | 1,497 | 1.05 (0.98-1.13), p=0.181 | 0.94 (0.86-1.03), p=0.160 | 0.95 (0.87-1.04), p=0.239 | 0.97 (0.88-1.08), p=0.616 |
| Q5 (≥ 32.49) | 40.66 | 20,365 | 1,622 | 1.14 (1.06-1.23), p=0.001 | 0.90 (0.80-1.02), p=0.089 | 0.92 (0.82-1.03), p=0.141 | 0.96 (0.83-1.11), p=0.598 |
|  |  |  |  | p _for trend_<0.001 | p _for trend_=0.174 | p _for trend_=0.258 | p _for trend_=0.799 |
| P-MUFAs |  |  |  |  |  |  |  |
| Q1 (< 4.88 ) | 3.65 | 20,414 | 1,460 | Reference group | Reference group | Reference group | Reference group |
| Q2 (≥ 4.88 to < 7.18) | 6.01 | 20,340 | 1,397 | 0.94 (0.87-1.01), p=0.092 | 0.94 (0.88-1.02), p=0.135 | 0.95 (0.88-1.02), p=0.176 | 0.96 (0.89-1.03), p=0.262 |
| Q3 (≥ 7.18 to < 9.89 ) | 8.44 | 20,393 | 1,361 | 0.90 (0.83-0.97), p=0.005 | 0.87 (0.80-0.94), p=0.001 | 0.87 (0.80-0.94), p=0.001 | 0.89 (0.81-0.96), p=0.004 |
| Q4 (≥ 9.89 to < 14.16 ) | 11.67 | 20,355 | 1,452 | 0.94 (0.87-1.01), p=0.091 | 0.87 (0.80-0.95), p=0.001 | 0.88 (0.81-0.95), p=0.002 | 0.90 (0.82-0.99), p=0.026 |
| Q5 (≥ 14.16) | 18.44 | 20,335 | 1,491 | 0.94 (0.88-1.02), p=0.128 | 0.82 (0.75-0.91), p<0.001 | 0.83 (0.75-0.91), p<0.001 | 0.88 (0.77-1.00), p=0.043 |
|  |  |  |  | p _for trend_=0.397 | p _for trend_<0.001 | p _for trend_<0.001 | p _for trend_=0.069 |
| A-MUFAs |  |  |  |  |  |  |  |
| Q1 (< 6.63 ) | 4.95 | 20,388 | 1,280 | Reference group | Reference group | Reference group | Reference group |
| Q2 (≥ 6.63 to < 9.64) | 8.11 | 20,393 | 1,336 | 1.06 (0.98-1.14), p=0.141 | 1.01 (0.94-1.10), p=0.729 | 1.02 (0.94-1.10), p=0.638 | 1.02 (0.94-1.10), p=0.691 |
| Q3 (≥ 9.64 to <13.08 ) | 11.23 | 20,338 | 1,323 | 1.04 (0.96-1.12), p=0.374 | 0.97 (0.89-1.05), p=0.456 | 0.98 (0.90-1.06), p=0.618 | 0.97 (0.89-1.06), p=0.556 |
| Q4 (≥ 13.08 to < 18.48) | 15.33 | 20,358 | 1,533 | 1.19 (1.10-1.28), p<0.001 | 1.05 (0.96-1.14), p=0.319 | 1.06 (0.97-1.15), p=0.21 | 1.05 (0.95-1.15), p=0.353 |
| Q5 (≥ 18.48 ) | 23.76 | 20,360 | 1,689 | 1.31 (1.21-1.41), p<0.001 | 1.06 (0.95-1.18), p=0.287 | 1.08 (0.97-1.20), p=0.185 | 1.05 (0.92-1.20), p=0.487 |
|  |  |  |  | p _for trend_<0.001 | p _for trend_=0.190 | p _for trend_=0.118 | p _for trend_=0.400 |

Abbreviations: SFAs, saturated fatty acids; PUFAs, polyunsaturated fatty acids; MUFAs, monounsaturated fatty acids; TFAs, trans-fatty acids; P-MUFAs, MUFAs from plant; A-MUFAs, MUFAs from animal; Q, quintile.

^b^ Additionally adjusted for race (non-Hispanic White vs. Other), body mass index (continuous), education (≤ high school vs. ≥ some college), smoking status (never vs. former ≤ 15 years since quit vs. former > 15 years since quit vs. former year since quit unknown vs. current smoker ≤ 1 pack per day vs. current smoker > 1 pack per day vs. current smoker intensity unknown), total energy intake (continuous), alcohol drinking status (never vs. former vs. current), study center (categorical), marital status (married vs. not married), randomization arm (screening group vs. control group), aspirin use (yes vs. no), history of hypertension (yes vs. no), history of diabetes (yes vs. no), vegetables intake (continuous), and fruit intake (continuous).

^c^ Further adjusted for history of stroke (yes vs. no) and history of heart attack (yes vs. no).

^d^ Additionally adjusted for other remaining fatty acids.
