# Supplementary figures and images for: Associations of Dietary Fat Intake With Mortality From All Causes, Cardiovascular Disease, and Cancer: A Prospective Study
Source: Front Nutr. 2021 Aug 9;8:701430. doi: 10.3389/fnut.2021.701430 (PMC8380819; doi:10.3389/fnut.2021.701430)

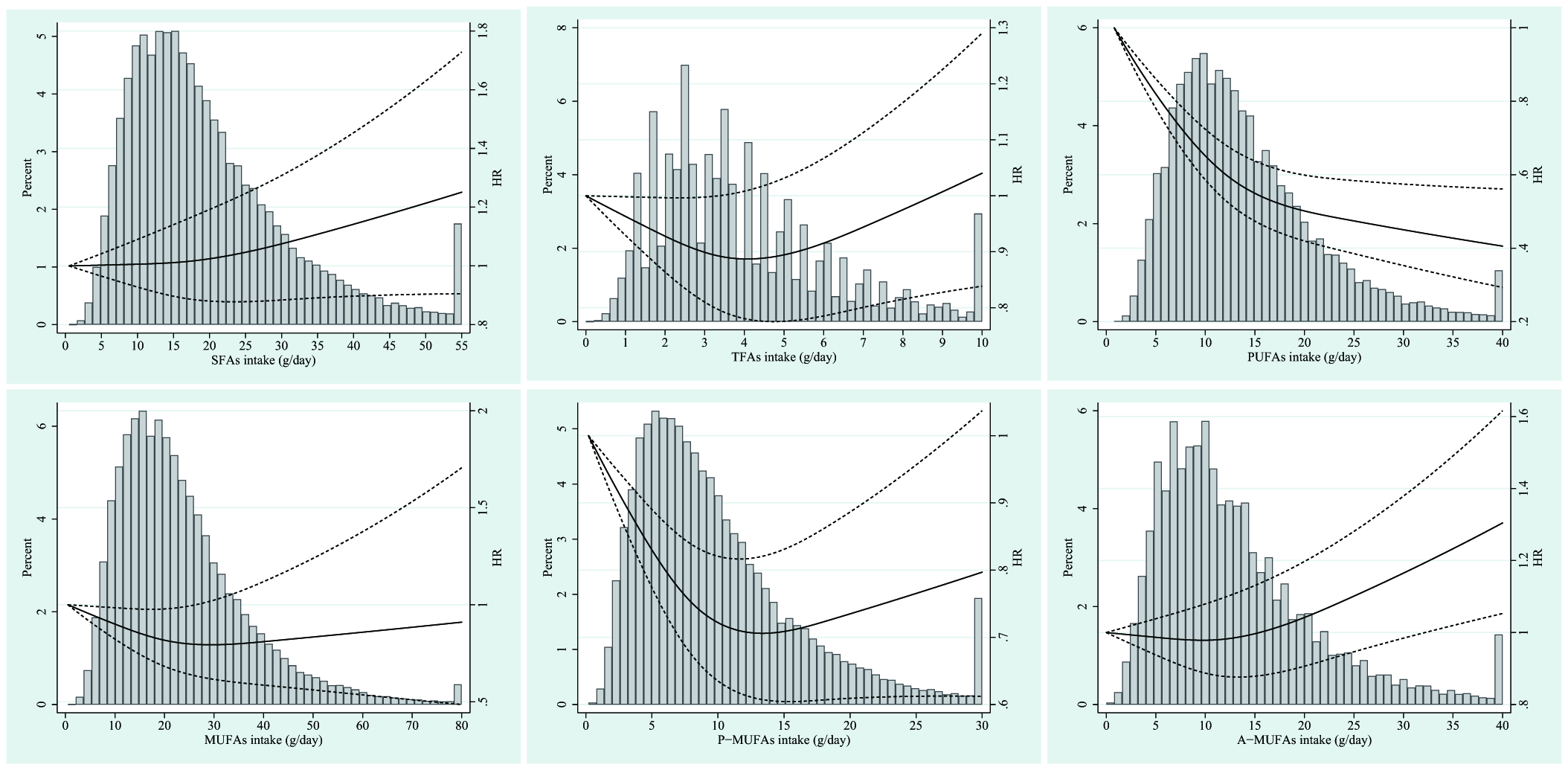

Supplement: Supplementary Figure 1 — Dose-response analyses for the association between intake of specific dietary fat and cardiovascular disease (CVD) mortality were performed using restricted cubic spline model. Solid lines represent point estimates and dashed lines represent 95% confidence intervals (CIs). Multivariable Hazard ratios (HRs) were calculated by restricted cubic spline regression (using three knots at 10th, 50th, and 90th percentiles) adjusting for age, gender, race, body mass index, education, smoking status, total energy intake, alcohol drinking status, study center, marital status, randomization arm, aspirin use, history of hypertension, history of diabetes, history of stroke, history of heart attack, vegetables intake, fruit intake, and other remaining fatty acids. The histograms show the percentage of participants (left y axis) belonging to each level of specific dietary fat. [file Image_1.TIF]

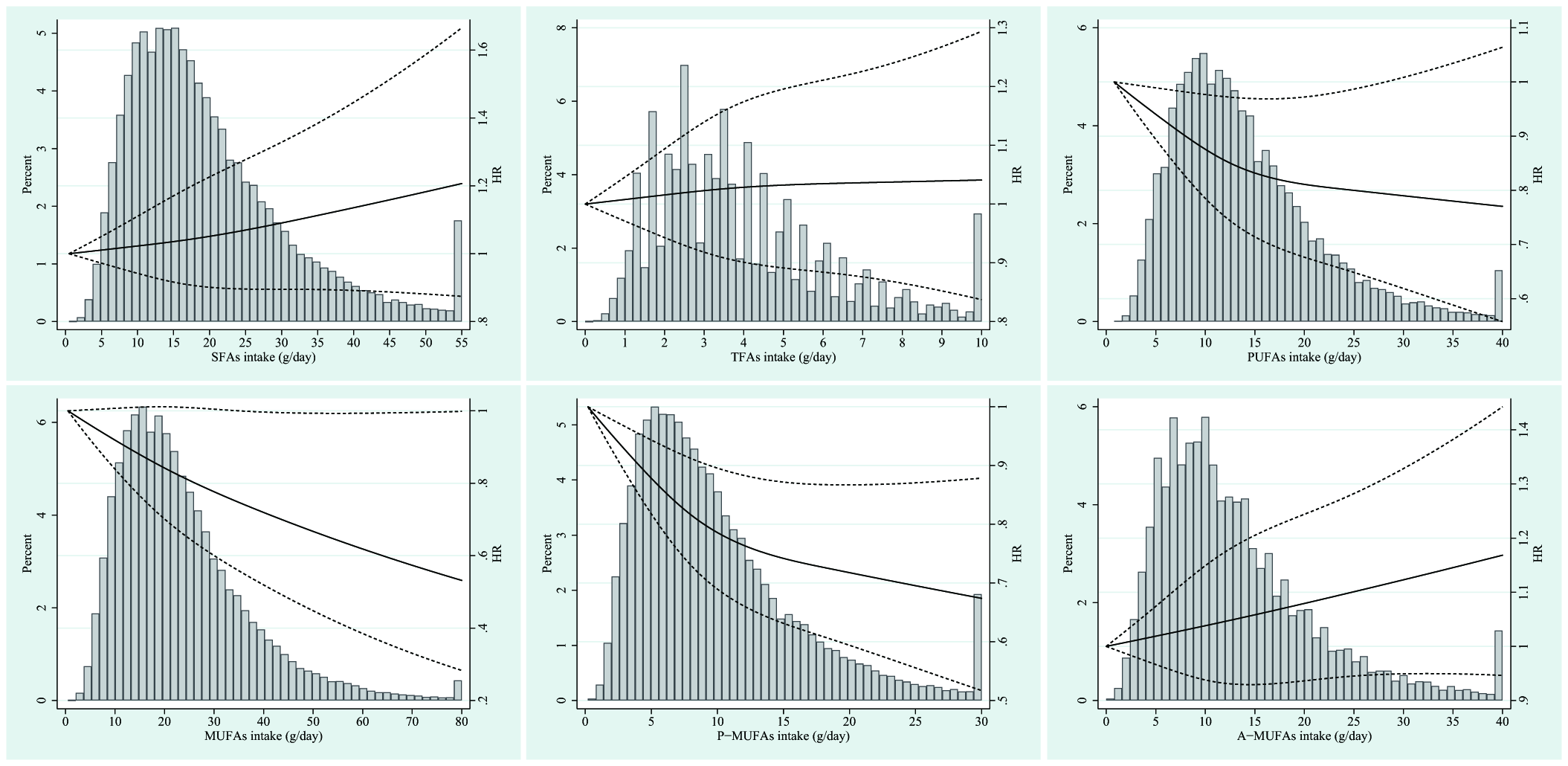

Supplement: Supplementary Figure 2 — Dose-response analyses for the association between intake of specific dietary fat and cancer mortality were performed using restricted cubic spline model. Solid lines represent point estimates and dashed lines represent 95% confidence intervals (CIs). Multivariable Hazard ratios (HRs) were calculated by restricted cubic spline regression (using three knots at 10th, 50th, and 90th percentiles) adjusting for age, gender, race, body mass index, education, smoking status, total energy intake, alcohol drinking status, study center, marital status, randomization arm, aspirin use, history of hypertension, history of diabetes, history of stroke, history of heart attack, vegetables intake, fruit intake, and other remaining fatty acids. The histograms show the percentage of participants (left y axis) belonging to each level of specific dietary fat. [file Image_2.TIF]
